# A Gestational Profile of Placental Exosomes in Maternal Plasma and Their Effects on Endothelial Cell Migration

**DOI:** 10.1371/journal.pone.0098667

**Published:** 2014-06-06

**Authors:** Carlos Salomon, Maria Jose Torres, Miharu Kobayashi, Katherin Scholz-Romero, Luis Sobrevia, Aneta Dobierzewska, Sebastian E. Illanes, Murray D. Mitchell, Gregory E. Rice

**Affiliations:** 1 University of Queensland Centre for Clinical Research, Centre for Clinical Diagnostics, Royal Brisbane and Women's Hospital, Queensland, Australia; 2 Department of Obstetric and Gynaecology, Faculty of Medicine, Universidad de los Andes, Santiago, Chile; 3 Cellular and Molecular Physiology Laboratory (CMPL), Division of Obstetrics and Gynaecology, School of Medicine, Faculty of Medicine, Pontificia Universidad Católica de Chile, Santiago, Chile; VU University Medical Center, Netherlands

## Abstract

Studies completed to date provide persuasive evidence that placental cell-derived exosomes play a significant role in intercellular communication pathways that potentially contribute to placentation and development of materno-fetal vascular circulation. The aim of this study was to establish the gestational-age release profile and bioactivity of placental cell-derived exosome in maternal plasma. Plasma samples (n = 20 per pregnant group) were obtained from non-pregnant and pregnant women in the first (FT, 6–12 weeks), second (ST, 22–24 weeks) and third (TT, 32–38 weeks) trimester. The number of exosomes and placental exosome contribution were determined by quantifying immunoreactive exosomal CD63 and placenta-specific marker (PLAP), respectively. The effect of exosomes isolated from FT, ST and TT on endothelial cell migration were established using a real-time, live-cell imaging system (Incucyte). Exosome plasma concentration was more than 50-fold greater in pregnant women than in non-pregnant women (p<0.001). During normal healthy pregnancy, the number of exosomes present in maternal plasma increased significantly with gestational age by more that two-fold (p<0.001). Exosomes isolated from FT, ST and TT increased endothelial cell migration by 1.9±0.1, 1.6±0.2 and 1.3±0.1-fold, respectively compared to the control. Pregnancy is associated with a dramatic increase in the number of exosomes present in plasma and maternal plasma exosomes are bioactive. While the role of placental cell-derived exosome in regulating maternal and/or fetal vascular responses remains to be elucidated, changes in exosome profile may be of clinical utility in the diagnosis of placental dysfunction.

## Introduction

The placenta plays a pivotal role in orchestrating maternal adaptation to pregnancy and synthesises and releases pregnancy-associated autacoids that influence fetal, placental and maternal metabolism [1]. In addition to soluble endocrine mediators, placental cells (including cytotrophoblasts, syncytiotrophoblast and placental mesenchymal stem cells) also release extracellular vesicles (ECVs – including: apoptotic bodies/vesicles (100–600 nm); shed microvilli (>400 nm); microvesicles (0.1–2 µm); and exosomes (30–100 nm)[2]) that may also modify maternal physiology and fetal development [3]. Indeed, over the past five years, our understanding of how cells communicate with each other, in health and disease, has undergone a paradigm shift with the recognition of the role of exosomes in inter-cellular signaling [4]. The role of placenta-derived exosomes in maternal adaptation to pregnancy remains to be established.

Exosomes are cell-derived bioactive vesicles, secreted by exocytosis and reflect aspects of the physiological state and function of the originating cells, including the human placenta [5], [6]. In addition, the release and content of these nanovesicles can be affected by the extracellular micro-environment.[7]. Exosomes are characterised as spherical bilayer lipid vesicles of 30–100 nm diameter when imaged using cryosection electron microscopy. The exosomal lipid bilayer contains relatively high concentrations of cholesterol, sphingomyelin, ceramide and detergent resistant membrane domains (lipid rafts) making these vesicles extremely stable.[8]. Exosomes regulate intracellular pathways by sequestering signalling molecules from the cytoplasm by reducing their bioavailability or preventing their packaging and release via exocytosis.[9]. They contain mRNA, miRNA [10], cell-surface receptors [11] and protein mediators [12] that can interact with either adjacent or distal cells to reprogram their phenotype and regulate cell function [13]. Available data suggest that the release of exosomes represents an important and common mode of cell-to-cell communication [14] under both normal and pathological conditions [15], [16].

Previous studies have identified the presence of exosomal proteins released from first trimester and term placenta, including molecules with immunological functions such as HLA-G and B7 family immunomodulators [17]; soluble MHC class I chain-related protein A and B [18]; syncytin-1 expression [19]. Available data support a role for placenta-derived ECVs in immunosuppression and maintenance of the fetal allograft.

Placenta-derived ECVs (including exosomes and microvesicles) have been identified in maternal plasma during normal pregnancy [20]–[22]. They contain placenta-specific proteins and miRNA, thus, may be differentiated from maternally-derived exosomes [23]–[25]. While the release of ECVs has been reported to change in association with complications of pregnancy, such as preeclampsia, the identity of ECV involved has yet to be unequivocally established and/or well characterised [21], [22], [26]–[28]. Studies completed to date provide persuasive evidence that placental cell-derived exosomes play a significant role in intercellular communication pathways that potentially contribute to placentation and development of maternal-fetal vascular exchange. Furthermore, the release and composition of these ECVs during normal pregnancies remain to be fully elucidated. We hypothesise that during normal, uncomplicated pregnancy the concentration of placenta-derived exosomes in maternal blood increases during pregnancy and correlates with uterine blood flow and placental weight at delivery. Furthermore, we suggest placenta-derived exosomes mediate maternal vascular adaptation to pregnancy. As such, the analysis of placenta-derived exosomes in maternal blood may represent a clinically useful, non-invasive test for placental function and/or dysfunction.

The specific aims of this study were to establish pregnancy-associated changes in: the concentration, content and bioactivity of placenta-derived exosomes in maternal blood during pregnancy from normal healthy women.

## Materials and Methods

### Study group and samples

A time-series experimental design was used to established pregnancy-associated change in exosome concentration, content and bioactivity in maternal blood. Women were recruited, with informed, written consent, by research midwives from the Clinica Davila, Santiago, Chile. Serial blood samples (BD Vacutainer PLUS Tubes. EDTA) were collected from normal, healthy pregnant women (n = 20 women sampled three times during pregnancy) during first trimester (6–12 weeks), second trimester (22–24 weeks) and third trimester (32–38 weeks) of pregnancy. Human plasma samples were obtained in accordance with the Declaration of Helsinki and approved by the ethics committee of The University of Queensland.Tissue collection was approved by the Human Research Ethics Committees of the Royal Brisbane and Women's Hospital, and the University of Queensland (HREC/09/QRBW/14). Doppler ultrasound examinations of the umbilical artery (left and right arteries) were performed across the pregnancy (n = 49, 11 missing data values for pulsatility index across the pregnancy). Plasma samples were stored at −80°C until analysed (samples were not stored for more than three months). All experimental procedures were conducted within an ISO17025 accredited (National Association of Testing Authorities, Australia) research facility. All data were recorded within a 21 CRF part 11 compliant electronic laboratory notebook (Irisnote, Redwood City, CA, USA).

### Isolation of exosomes from maternal circulation

Exosomes were isolated from plasma (1 ml) as previously described. [29] In brief, plasma was diluted with an equal volume of PBS (pH 7.4) and centrifuged at 2,000×g for 30 min at 4°C (Sorvall, high speed microcentrifuge, fixed rotor angle: 90°, Thermo Fisher Scientific Ins., Asheville, NC, USA,). The 2000 g supernatant fluid was then centrifuged at 12,000×g for 45 min at 4°C (Sorvall, high speed microcentrifuge, fixed rotor angle: 90°). The resultant supernatant fluid (2 ml) was transferred to an ultracentrifuge tube (Sorvall ultracentrifuge tubes) and centrifuged at 200,000×g for 75 min (Sorvall, T-8100, fixed angle ultracentrifuge rotor The pellet was suspended in PBS (30 ml) and filtered through a 0.22 µm filter (Steritop™, Millipore, Billerica, MA, USA) and then centrifuged at 200,000×g for 75 min. The 200,000 g pellet was resuspended in 2.5 M sucrose solution (4 ml) and added at the bottom of the ultracentrifuge tubes. A continuous sucrose gradient (25 ml; 0.25–2.5 M) was made above 4 ml of exosomes using a Hoefer SG30 gradient maker (GE Healthcare, NSW, Australia) and centrifuged at 110,000 g for 20 h (Sorvall, SureSpinTM 630/360, Swinging-Bucket ultracentrifuge rotor). Fractions (10 in total, 3 ml each) were collected with an 18-G needle and the density of each fraction was determined using the refraction index with OPTi digital refractometer (Bellingham+Stanley Inc., Lawrenceville, GA, USA). Fractions (3 ml each) were diluted in PBS (60 ml) and then centrifuged at 200,000×g for 70 min. The 200,000 g pellet was resuspended in 50 µl PBS and stored at −80°C. Protein concentration was determined by the DC Protein Assay (BIO-RAD, Hercules, CA, USA). Briefly, exosome samples (5 µl) were prepared by adding RIPA buffer (50 mM Tris, 1% Triton × 100, 0.1% SDS, 0.5% DOC, 1 mM EDTA, 150 mM NaCl, protease inhibitor) directly to exosomes suspended in PBS and sonicated at 37°C for 15 s three times to lyse exosome membrane and solubilize the proteins. Bovine serum albumin (BSA) diluted in RIPA buffer and PBS mixture (1∶1) was prepared as protein standards (0, 200, 400, 600, 800, 1,000, 1,500 µg/mL). Standards and samples (exosomes) were transferred to 96-well plates. Alkaline copper tartrate solution (BIO-RAD, USA) and dilute Folin Reagent (BIO-RAD, USA) were added to the samples and incubated for 15 min. The absorbance was read at 750 nm with the Paradigm Detection Platform (Beckman Coulter, USA).[7]

### Western blot (CD63, CD81, CD9 and PLAP)

Protein from each sucrose gradient fraction (10 in total) obtained after exosome isolation were separated by SDS polyacrylamide gel electrophoresis[7], [30] using a XCell SureLock electrophoresis system (Life Technologies), transferred to Immobilon-FL polyvinylidene difluoride membranes (Millipore, Billerica, MA, USA) and probed with primary mouse monoclonal anti-CD63 (1∶2,000 ab8219, Abcam, Sapphire Bioscience Pty Ltd, NSW, Australia), anti-CD81 (1∶1,500 MAB6435, Abnova, Tapei City, Taiwan) and anti-CD9 (1∶1,500 ab2215, Abcam, Sapphire Bioscience) as previously described exosome enriched markers. [7], [18], [29], [30] Membranes were washed in Tris buffer saline (pH 7.6) and incubated (1 h) in TBST/0.2% BSA containing horseradish peroxidase–conjugated goat anti-mouse antibody. Proteins were detected by enhanced chemiluminescence with using a SRX-101A Tabletop Processor (Konica Minolta, Ramsey, NJ, USA). The exosomal fractions (from 1.126 to 1.187 g/ml) were pooled (*defined as exosomes*) and placental alkaline phosphatase (PLAP) protein abundance was determined by Western blot using primary polyclonal antibody anti-PLAP (1∶1,000 ab96588, Abcam, Sapphire Bioscience) using a GS-800 Calibrated Densitometer (Bio-Rad Laboratories).

### Transmission electron microscopy

Exosome pellets (as described above, 30 µg protein) were fixed in 3% (w/v) glutaraldehyde and 2% paraformaldehyde in cacodylate buffer, pH 7.3. Exosome samples were then applied to a continuous carbon grid and negatively stained with 2% uranyl acetate. The samples were examined in an FEI Tecnai 12 transmission electron microscope (FEI, Hillsboro, Oregon, USA) in the Central Analytical Research Facility, Institute for Future Environments, Queensland University of Technology (QUT) (see Acknowledgements).

### Nanoparticle tracking analysis (NTA)

NTA measurements were performed using a NanoSight NS500 instrument (NanoSight NTA 2.3 Nanoparticle Tracking and Analysis Release Version Build 0033) following the manufacturer's instructions. The NanoSight NS500 instrument measured the rate of Brownian motion of nanoparticles and consists in a light scattering system that provides a reproducible platform for specific and general nanoparticle characterization ((NanoSight Ltd., Amesbury, United Kingdom). Samples were processed in duplicate and diluted with PBS over a range of concentration for to obtain between 10 and 100 particles per image (optimal ∼50 particles x image) before the analysis with NTA system. Samples were added into the chamber (temperature: 25°C and viscosity: 0.89 cP) and the camera level set to obtain and image that has sufficient contrast to clearly identified particles while minimizing background noise a video recording (camera level: 10 and capture duration: 60 s). After the capture videos (2 videos per sample) were processed and analysed. A combination of high shutter speed (600) and gain (250) followed by manual focusing enables optimum visualization of a maximum number of vesicles. We included a minimum of 200 tracks completed per video in duplicate. NTA post acquisition settings were optimized and kept constant between samples (Frames Processed: 1496 of 1496, Frames per Second: 30, camera shutter: 20 ms; Calibration: 139 nm/pixel, Blur: 3×3; Detection Threshold: 10; Min Track Length: Auto; Min Expected Size: Auto), and each video was then analyzed to give the mean, mode, and median particles size together with an estimate of the number of particles. An Excel spreadsheet (Microsoft Corp., Redmond, Washington) was also automatically generated, showing the concentration at each particle size.

### Quantification of placental cell-derived exosome

The concentration of exosomes in maternal circulation is expressed as total immunoreactive exosomal CD63 (ExoELISA, System Biosciences, Mountain View, CA). Briefly, 10 µg of exosomal protein was immobilised in micro-titer plate wells and incubated over night (binding step). Plates were washed three times for 5 min using a wash buffer solution and then incubated with exosome specific primary antibody (CD63) at room temperature (RT) for 1 h with shaking. Plates were washed and incubated with secondary antibody (1∶5000) at RT 1 h with shaking. Plates were washed and incubated with Super-sensitive TMB ELISA substrate at RT for 45 min with shaking. The reaction was terminated using Stop Buffer solution. Absorbance was measured at 450 nm. The number of exosomes/ml, (ExoELISA kit) was obtained using an exosomal CD63 standard curve calibrated against nanoparticle tracking data (*i.e.* number of exosomes, NanoSight).

For placental cell-derived exosomes, the concentration of exosomal PLAP was quantified using a commercial ELISA kit (MBS701995, San Diego, CA, USA) according to manufacturer's instructions. Briefly, 10 µg of exosomal protein was added to each well of a microtitre plate and incubated at 37°C for 30 min. Plates were washed three times with shaking for 20 s and then 50 µl of HRP-conjugate was added to each well and incubated at 37°C for 20 min. Plates were washed and incubated with 50 µl of substrate A and 50 µl of substrate B at 37°C for 15 min. The incubation was terminated using 50 µl of stop solution at RT for 2 min with shaking. Absorbance was measured at 450 nm.

### Endothelial cells isolation

Umbilical cords were collected immediately after delivery from 6 full-term normal pregnancies from the Royal Brisbane and Women's Hospital (RBWH, Brisbane, Australia). The investigation conforms to the principles outlined in the Declaration of Helsinki and institutional ethical regulations. Human umbilical vein endothelial cells (HUVEC) were use to determine the bioactivity of exosomes isolated from maternal plasma. HUVEC primary cultures (37°C, 5% CO_2_) were isolated by enzymatic digestion using Collagenase Type II (Gibco Life Technologies, Carlsbad, CA) as previously described.[31] Cells were cultured in primary culture medium (PCM) containing 2% exosomes-depleted FBS (culture media was depleted of the contaminating exosomes using the same protocol for exosome isolation described previously and exosome-free culture media medium was confirmed by electron microscopy) for 24 h before experiments.

### Migration assay

To assess the effect of exosomes on endothelial cell, HUVEC were cultured in 96 well (Corning Life Science, Tewksbury, MA, USA) according to the manufacturer's instructions and visualised using a real-time cell imaging system (IncuCyte live-cell ESSEN BioScience Inc, Ann Arbor, Michigan, USA). Before experiments, HUVEC were cultured in PCM supplemented with 0.2% exosome-depleted FBS in 96-well culture plates (Corning Life Science) according to the manufacturer's instructions for 18–24 h. Cells were imaged every hour to monitor treatment-induced cell migration, confluence and morphologic changes. Cell migration was assessed by scratch assays, in which, HUVEC were grown to confluence and then a scratch was made using a 96-pin WoundMaker. For to prepare the treatments (*i.e.* exosomes), exosome enriched fraction were mixed using the same amount of protein from each patient (n = 20 per group). The wells were washed with PBS to remove any debris and incubated in the absence (-exo) or presence of mixed exosomes (100 µg exosomal proteins per ml of plasma) isolated from maternal plasma during first, second and third trimester of pregnancy and non-pregnant women (n = 12, *i.e.* 12 independent experiment in duplicate). Wound images were automatically acquired and registered by the IncuCyte software system. Typical kinetic updates were recorded at 2 h intervals for the duration of the experiment (24 h). The data were then analyzed using an integrated metric: Relative Wound density as previously described. [7] Vascular Endothelial Growth Factor human (VEGF, 300 ng/ml; Sigma-Aldrich) was used as a positive control for cell migration.

### Statistical analysis

Data are presented as mean ± SEM, with n = 20 different patients per group (*i.e.* first, second and third trimester). The effects of gestational age on plasma exosome number, exosomal protein and PLAP concentrations were assessed using ANOVA, with variance partitioned between, gestational age and subject. Statistical differences between groups were identified by *post hoc* analyses (multiple-comparison Bonferroni correction test.) Two group means were statistically assessed by Student's t-test. Spearman's correlation was used to assess the relationship between pulsatility index and gestational age. Statistical significance was defined as at least *p<0.05.* Statistical analyses were preformed using commercially available packages (Stata 11, StatCorp, College Station, Texas USA and Prism 6, GraphPad Inc, La Jolla, CA 92037 USA).

## Results

### Patients

All pregnant women included in this study were singleton, normotensive and without intrauterine infection or any other medical or obstetric complications. Mean maternal age was 27.83±6.1 years and BMI averaged 24.95±4.2 kg/m^2^ (Table 1). Placental weight at delivery averaged 595±96 g. Fetal weight at birth was 3435±459 g and the ratio male/female was 0.53 (7 male and 13 female newborn). The uterine arterial Doppler (left and right) analysis across the pregnancy showed overlapping outcomes. The average values for pulsatility index decreased progressively from the first trimester across the normal pregnancy (Table 1).

**Table 1:** Clinical characteristics of patients and newborns.

Womens		
Variables	Pregnant (n = 20)	Non-pregnant (n = 9)
Age (years)	27.83±6.1 (18–36)	25.53±7.1 (20–30)
Weight (kg)	63.74±11.52 (50–92)	60.44±10.92 (55–70)
Height (cm)	158.7±6.35 (148–167)	155.6±5.35 (150–163)
BMI (Kg/m^2^)	24.95±4.2 (20–37)	26.86±5.4 (22–30)

Newborn variables	
Placental weight (grams)	595±96 (434–749)
Fetal weight (grams)	3435±459 (2725–4830)
Fetal sex (male/female)	7/13
Pregnant women (gestational age)	
First trimester (weeks)	8.2±2.3 (6.2–12.3)
Second trimester (weeks)	25.2±2.0 (22.3–28.4)
Third trimester (weeks)	33.6±2.6 (30.2–38.2)
Systolic/diastolic blood pressure (mm Hg)	
First trimester (6–12 weeks)	105/64±11/7 (90–120/50–80)
Second trimester (22–28 weeks)	109/63±11/8 (90–130/50–80)
Third trimester (30–38 weeks)	108/64±10/6 (90–120/50–80)
Uterine Artery Doppler	
Pulsatility index left uterine artery	
First trimester	1.72±0.65* (0.31–2.9)
Second trimester	0.84±0.13^†^ (0.51–1.0)
Third trimester	0.67±0.27 (0.28–1.4)
Pulsatility index right uterine artery	
First trimester	1.59±0.83* (0.54–3.2)
Second trimester	0.87±0.25^†^ (0.43–1.5)
Third trimester	0.58±0.22 (0.27–1.2)

Data are presented as mean ± SD (range). All pregnancies were singleton and pregnant women were normotensive, nonsmoking, non-alcohol or drug consuming, and without intrauterine infection or any other medical or obstetrical complications. In Pulsatility index left and right uterine artery, *P<0.05 versus values in second and third trimester; †*P*<0.05 versus values in third trimester.

### Exosome isolation and characterisation

**Figure 1:**
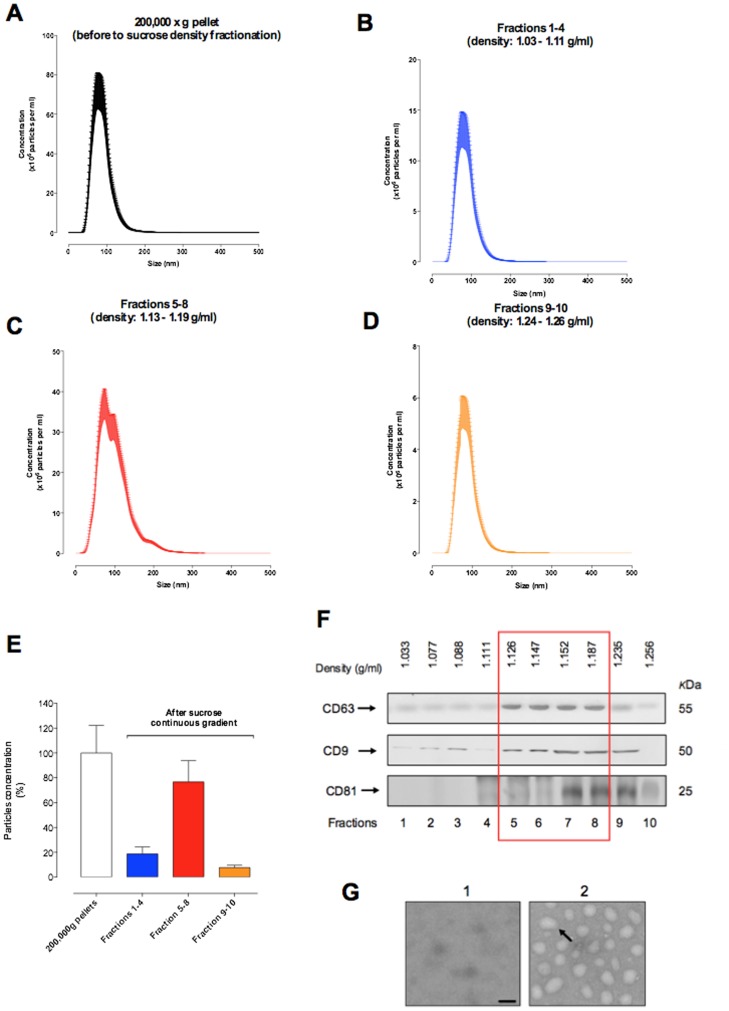
Characterisation of exosome from maternal circulation. Exosome were isolated from plasma of non-pregnant women and women uncomplicated pregnancies during first, second and third trimester by differential and buoyant density centrifugation (see Methods). (A–D) Representative vesicle size distribution isolated from maternal circulation across the pregnancy (first, second and third trimester) using a NanoSight NS500 instrument. Fractions 1 to 10, represent fractions collected after buoyant density centrifugation. (A) 200,000×g pellet (before sucrose density fractionation); (B) fractions 1–4; (C) fractions 5–8 enriched exosome population; (D) fractions 9–10. (E) percentage of vesicles before (white bar  = starting solution: 100%) and after (blue: fractions 1–4; red: fractions 5–8 and orange: fractions 9–10) of the exosome purification using a continuos sucrose gradient. (F) Representative Western blot for exosome enriched markers: CD63, CD9 and CD81. Exosome density is represented as red square. (G) Representative electron micrograph of 200,000×g supernatant (1) and exosome fractions (pooled enriched exosome population from fractions 5 to 8). In G, Scale bar 100 nm.

Exosome were isolated using the gold standard methods and purified by a sucrose continuous gradient. Nanoparticle tracking analysis showed that we obtained particles between 50 and 200 nm (Figure 1 A–D and table 2). In addition, after the sucrose continuous gradient we obtained an exosome enriched fractions corresponding ∼70% of the total particles after the ultracentrifugation (200,000×g) (Figure 1E). Maternal plasma exosomes isolated by differential and sucrose density gradient centrifugation were characterised by a buoyant density of 1.126 to 1.187 g/ml (fractions 5 to 8), the presence of CD63, CD81 and CD9 as assessed by Western blot (Figure 1F) and as ∼100 nm diameter particles when imaged by electron microscopy (Figure 1G).

**Table 2:** vesicles size distribution during pregnancy.

	Pregnant (trimester)			Non-pregnant
Condition	First	Second	Third	
200,000×g pellet*	110±69	120±75	115±65	125±50
Fractions 1–5	115±59	105±69	110±50	107±48
Fractions 5–8	98±39	100±60	92±40	97±55
Fractions 9–10	70±58	95±49	90±65	85±39

The size distribution of exosome preparations were analysed using a NanoSight NS500 system (NanoSight, Amesbury, UK) according to the manufacturer's instructions (see methods). Data are presented as mean ± SD. All preparation were analysed in duplicate (n = 20 per group) for pregnant women and non-pregnant women (n = 9). *Before to sucrose density fractionation.

### Gestational age variation in exosome concentration

**Figure 2:**
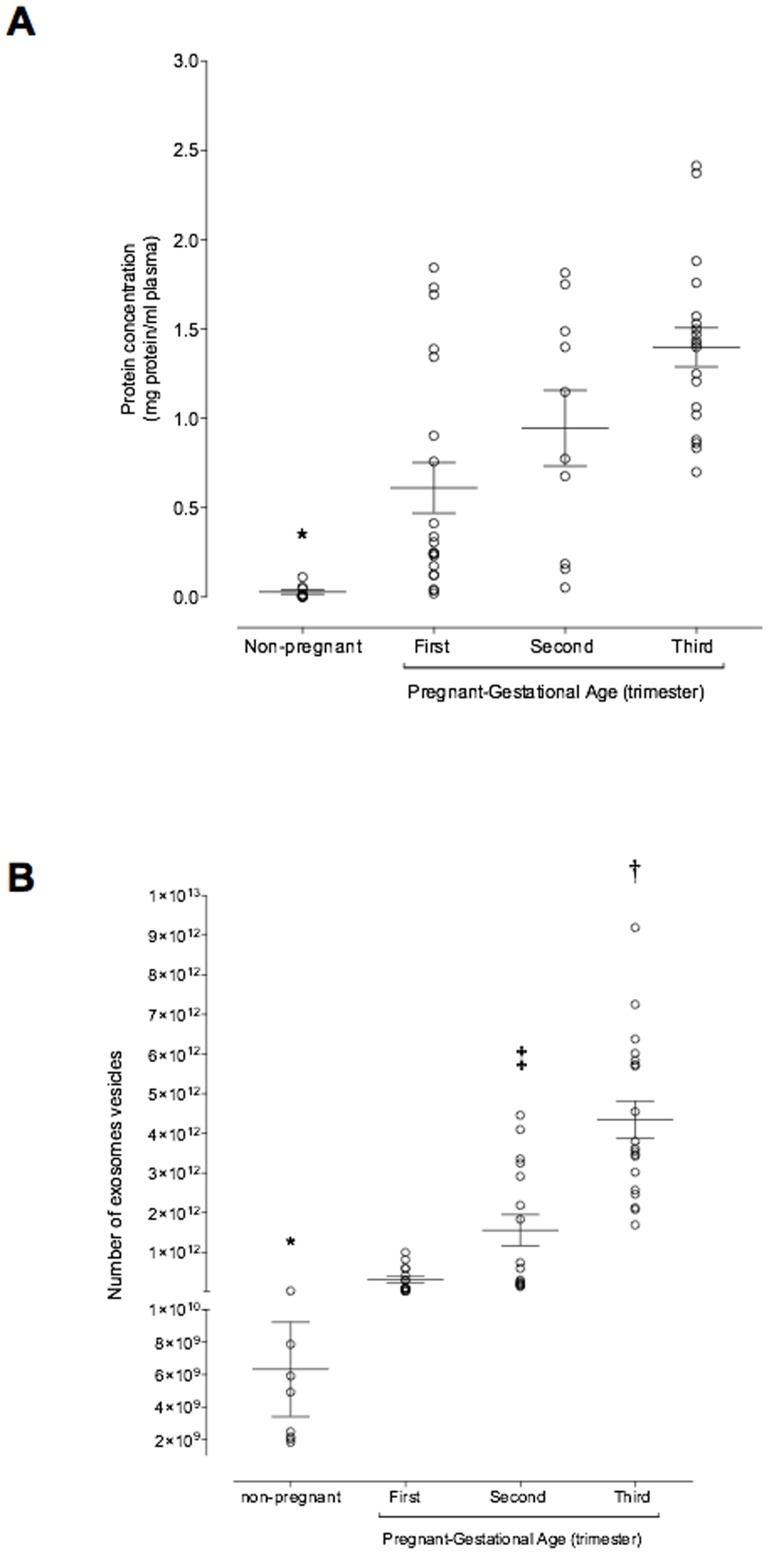
Exosome profiling across the pregnancy. Enriched exosomal protein population and number of exosomes were quantified in in peripheral plasma of women in the first, second and third trimester of pregnancy and non-pregnant women using a colorimetric assay and ELISA kit, respectively. (A) Plasma exosome concentration presented as mg exosomal protein per ml plasma and (B) Number of exosomes across the pregnancy. Data are presented as aligned dot plot and values are mean ± SEM. In A and B, *p<0.01 versus all; In B, ^†^p<0.05 versus ST trimester and ^‡^p<0.01 versus FT.

Pooled exosome-containing fractions (*i.e.* fractions 5 to 8) were further characterised by measuring total exosomal protein concentration. Plasma exosomal protein concentration in non-pregnant (n = 9) women averaged 0.03±0.01 mg protein/ml and was significantly lower than that observed in the plasma of women during first trimester (0.61±0.14 mg protein/ml, n = 20, p<0.001, Student's t-test). Plasma exosomal protein concentrations increased significantly during pregnancy and averaged 0.94±0.41 and 1.40±0.11 mg/ml in plasma in second and third trimester respectively (Figure 2A, ANOVA, p<0.003). No significant effect of subject on plasma exosomal protein concentrations was identified (p>0.6). Maternal plasma exosomal protein concentrations in first trimester were significantly less than that observed in second and third trimester (p<0.05, Fisher's least significant difference, pairwise comparison). No significant difference between second and third trimester group means was identified (p<0.05).

The number of exosomes (number of exosome vesicles, NEV) and exosomal PLAP concentration was quantified in serial samples of maternal plasma obtained during first, second and third trimester. In pregnant women, NEV increased significantly (ANOVA, p<0.001) with the progression of pregnancy. During second and third trimester, NEV was ∼ 4.8 and ∼13.5 fold higher compared with NEV in the first trimester of pregnancy, respectively (Figure 2B). Exosomes in plasma from non-pregnant women was significantly less (6.13×10^9^±2.13×10^9^ number of exosomes) compared to the number of exosomes in pregnant women across the gestation (data not shown). In addition, we identified a significant correlation between NEV and total exosomal protein concentration (Spearman's r = 0.56; p = 0.0001) (see Figure S1).

### Placenta-derived exosomes

**Figure 3:**
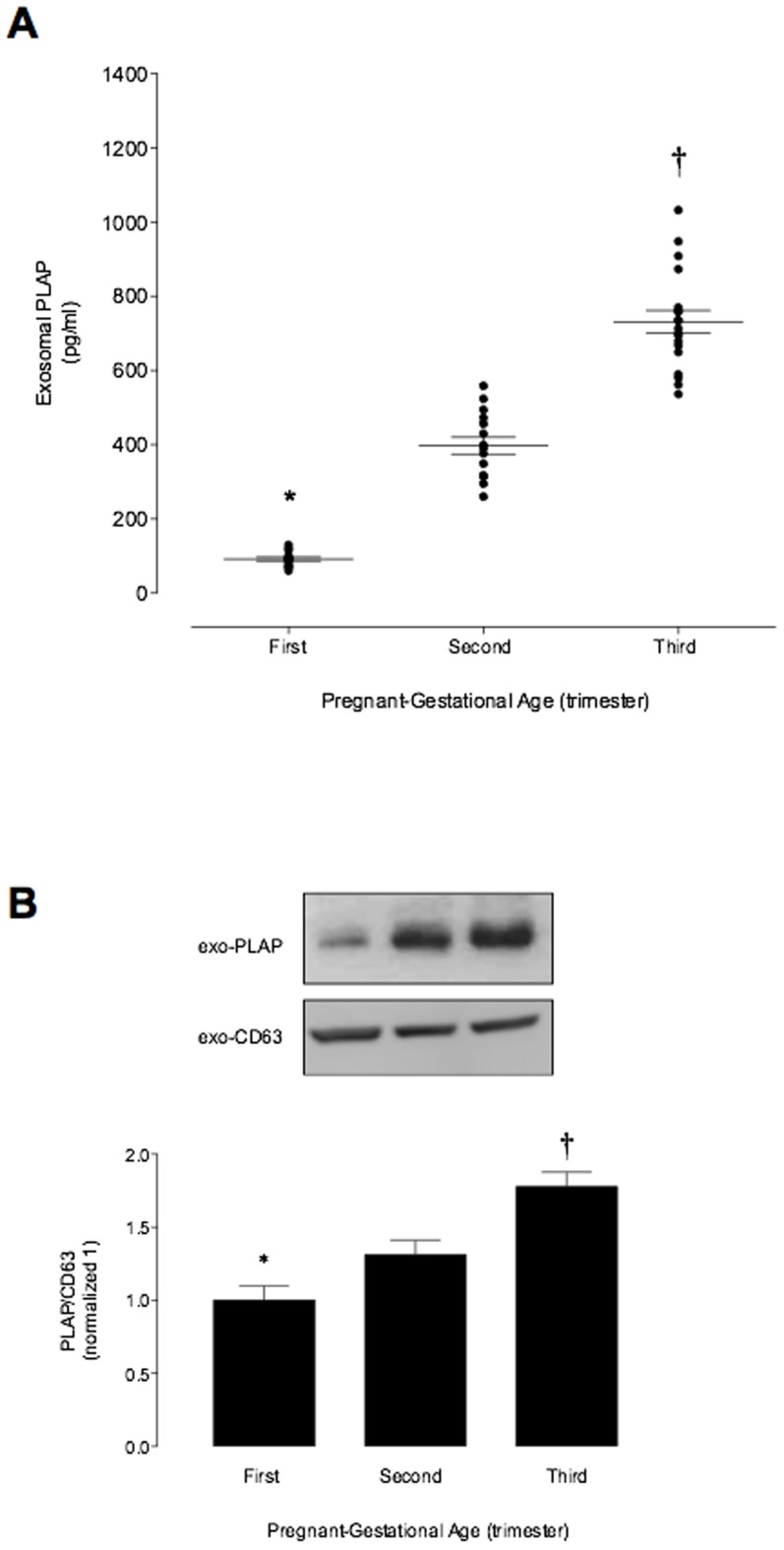
Placenta-derived exosomes profile during pregnancy. Enriched exosome vesicles were quantified in in peripheral plasma of women in the first, second and third trimester of pregnancy using an ELISA kit. (A) exosomal PLAP concentration across the normal pregnancy. (B) Same volume of enriched exosome pellet loaded and analyzed by Western Blot for PLAP and CD63 in exosomes (fractions 5 to 8 were pooled) from maternal plasma in the first, second and third trimester of pregnancy. Lowe panel: PLAP/CD63 ratio densitometries from data in top normalized to 1 (first trimester). Data are presented as aligned dot plot and values are mean ± SEM. In A and B, *p<0.01 versus ST and TT trimester; ^†^p<0.05 versus ST trimester.

The concentration of placenta-derived exosomes in maternal plasma (as indicated by exosomal PLAP) increased with gestational age: first trimester 99.8±5.3 pg/ml; second trimester 397±23; and third trimester 731±35 pg/ml (Figure 3A). Immunoreactive exosomal PLAP was not detectable in plasma of non-pregnant women (data not shown). Similar results were observed in the exosomal PLAP protein abundance (Western Blot) during the normal pregnancy (Figure 3B). No significant effects of fetal gender, maternal body max index (BMI), maternal age, maternal weight and maternal height on exosome number or exosomal PLAP were identified (data not shown).

### Exosomal PLAP and physiological correlates

**Figure 4:**
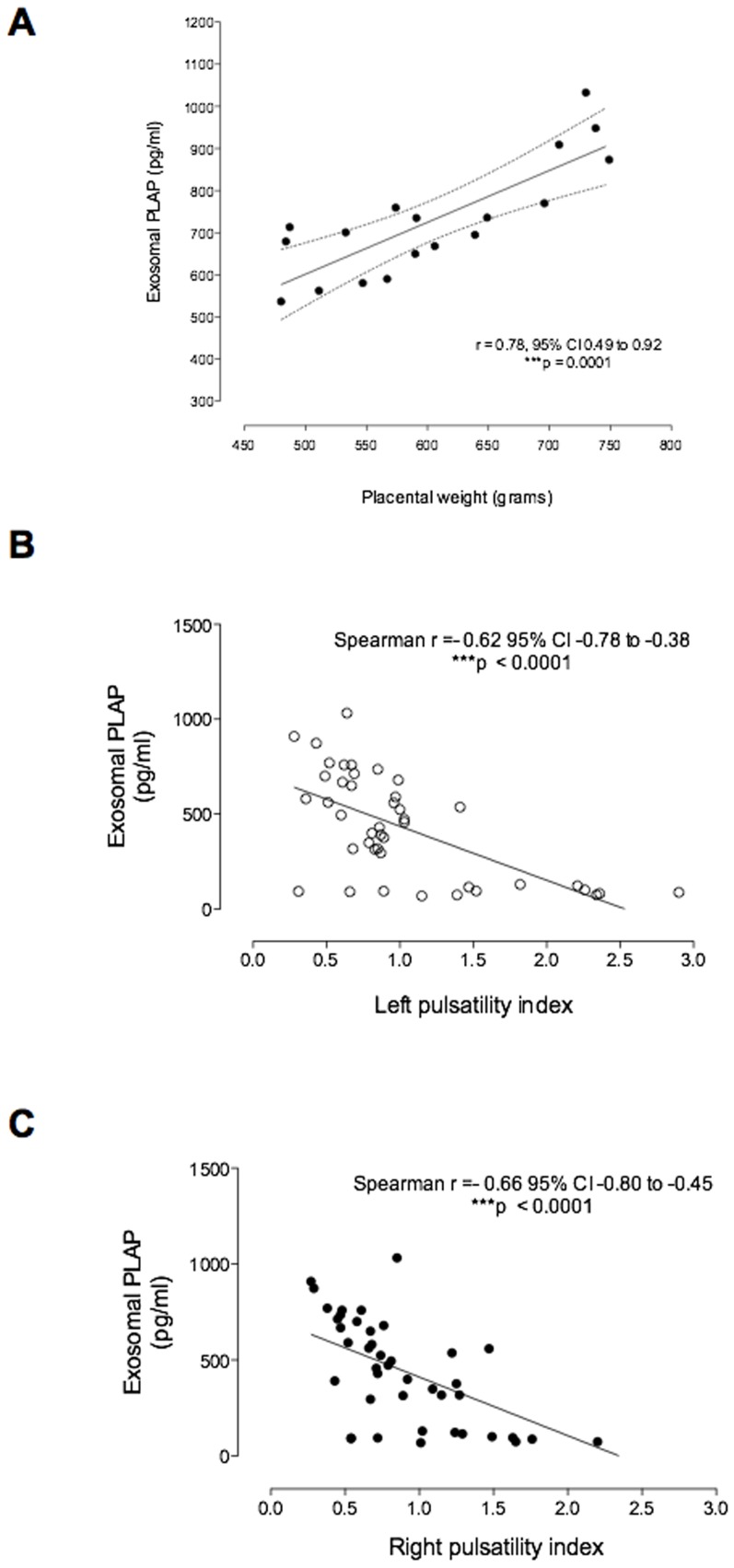
Contribution of placental-derived exosomes into maternal circulation. (A) Relationship between exosomal PLAP and NEV across the pregnancy. (B) Relationship between exosomal PLAP at TT of pregnancy and placental weight at delivery. (C and D) Relationship between exosomal PLAP and left and right Pulsatility Index (PI) across the pregnancy, respectively. Linear correlation (-). In A, n = 18 (2 missing data values). In B and C n = 49 (11 missing data values for PI across pregnancy).

At term, exosomal PLAP concentration in maternal plasma was strongly correlated with the placental weight (Spearman's r = 0.68; p = 0.0001) (Figure 4A). In addition, Uterine Doppler analysis displayed a negative correlation (Spearman's r = −0.62, p<0.0001) between mean pulsatility index and exosomal PLAP concentration across the pregnancy (Figure 4 B and C).

### Contribution of placental-derived exosomes to total exosomes present in plasma

**Figure 5:**
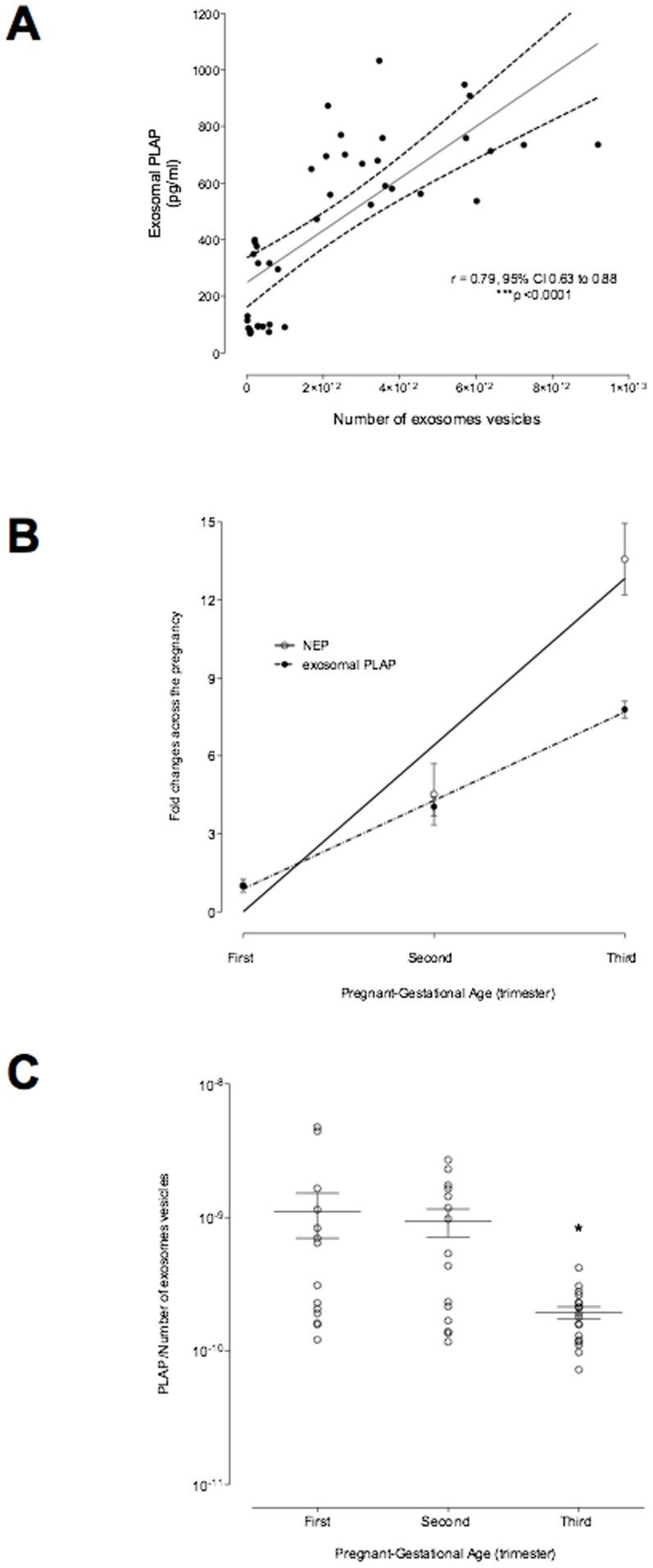
PLAP activity across the pregnancy. The specific exosomal PLAP activity (defined as PLAP concentration per number of exosome vesicles) was quantified in plasma of women in the first, second and third trimester. (A) Changes in NEV and specific placental exosomes into maternal circulation during normal pregnancy. (B) Ratio of specific placental exosome and NEV. IN B, *p<0.05 versus FTand ST trimester.

To estimates changes in the relative contribution of placental exosomes to total exosomes present in maternal plasma, PLAP content per exosome (PLAP ratio) was determined. Both exosomal PLAP concentration (pg/ml plasma) and total number of exosomes/ml plasma (NEV/ml plasma) increased throughout pregnancy and were significantly correlated (Spearman's r = 0.79, p<0.001; Figure 5A). NEV/ml accounted for ∼60% of the observed variation in exosomal PLAP (as assessed by linear regression analysis and the coefficient of determination  =  0.56, p<0.001). The fold change was similar during first and second trimester, however, NEV/ml increased independent of exosomal PLAP during third trimester (Figure 5B). Plasma PLAP ratio was constant during first and second trimester but decreased 4-fold during third trimester (Bonferroni's multiple comparison test, p<0.001) (Figure 5C). These data may represent a decrease in the release of PLAP^+ve^ exosomes from the placenta, increased release of exosomes from non-placental sources or a combination of both. The data obtained herein do not allow discrimination between these possible scenarios.

### Effect of exosomes on endothelial cell migration

**Figure 6:**
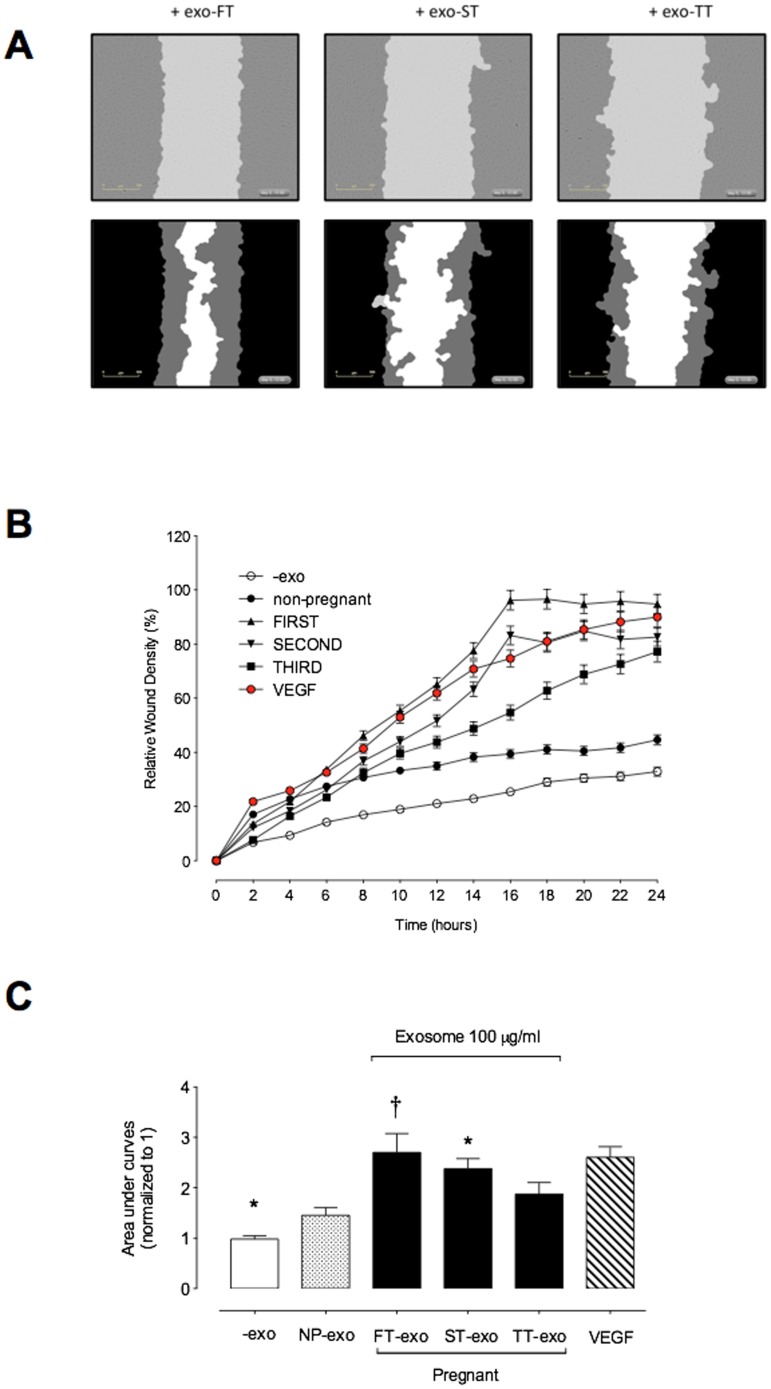
Maternal exosome effects on endothelial cells migration. HUVEC were grown to confluence in PCM and a wound was made using 96 well WoundMaker (see Methods). HUVEC Migration was measured in absence or presence of 100 ug/ml of exosomes from first, second and third trimester for 24 h. (A) Graphical representation from a showing the calculation of initial wound width (black) and graphical representation of cell migration (gray) at the midpoint of the experiment. (B) Time course of wound closure for HUVEC expressed as relative wound density (%). (C) Area under curves from data in B. Data represent an n = 12 well each point with 6 different cells culture (*i.e.* biological replicates) of HUVEC isolated from first trimester pregnancies (see methods). Values are mean ± SEM. In C, *p<0.05 versus all condition; ^†^ *P*<0.005 versus second and third trimester exosomes.

The effect of maternal circulation-derived exosomes isolated from first (FT-exo), second (ST-exo) and third (TT-exo) trimester of pregnancy on endothelial cell migration was determined using a real time cell imaging system (IncuCyte live-cell ESSEN BioScience Inc, Ann Arbor, Michigan, USA) and human umbilical vein endothelial cells (HUVEC). The effect of 100 µg exosomal protein/ml on HUVEC migration is presented in Figure 6 A, B and C. Representative photomicrographs of HUVEC wound closure with treatment (exo-FT, exo-ST and exo-TT) are presented in Figure 6A. The effect of exosomes on HUVEC migration is presented as relative wound density (percent) overtime (Figure 6B). Analysis of the area under the cell migration curves was used to assess the effect of gestational age on the bioactivity of maternal plasma exosomes. Maternal plasma exosomes isolated from FT, ST and TT increased HUVEC migration by: 2.7 fold; 2.3 fold; and 1.87 fold respectively when compared to cell migration without exosomes (-exo) (Figure 6C). In contrast, exosomes from non-pregnant women increases HUVEC migration ∼1.45 fold compared to cell migration without exosomes (-exo). The effect of exo-FT and exo-ST on HUVEC migration was significantly higher (p<0.05) versus the effect of exosomes from non-pregnant women. In addition, VEGF induced (∼2.9-fold) HUVEC migration to compare to control in absence of exosomes.

## Discussion

The aim of this study was to characterise pregnancy-associated changes in the concentration and bioactivity of exosomes present in maternal peripheral plasma. The data obtained in this study establish, that pregnancy is associated with a 50-fold increase in the concentration of exosomes in maternal plasma. In a longitudinal study, the concentration of placental exosomes in plasma increased during pregnancy and correlated with mean uterine artery blood flow (mean pulsatility index). At delivery, plasma concentration of placental exosomes correlated with placental weight. The study further established that exosomes present in maternal plasma are bioactive and promote cell migration. These data clearly establish that normal healthy pregnancy is characterised by the release of bioactive exosomes into maternal blood from as early as 6 weeks of gestation and that the concentration of placenta-specific exosomes is information of placental perfusion and growth. This study provides proof-of-principle and baseline data that may facilitate the early identification of dysfunctional placentation.

In this study, exosomes were isolated using differential and buoyant density centrifugation (the “gold standard method”) to obtain enriched exosome population. [29], [32] Nanovesicles isolated using this method, displayed a diameter between ∼40–120 a buoyant density on sucrose gradient of 1.21–1.192 g/m and were positive for the exosome markers, CD63, CD9 and CD81. These data are characteristic of exosomes and consistent with previously published data. [2], [7], [33]

The data obtained in this study establish that pregnancy is associated with a dramatic increase in the number of exosomes circulating in maternal blood. The concentration of exosomal protein in first trimester plasma was 50-fold greater than that observed in non-pregnant women. Maternal plasma exosomal protein concentrations increased a further two-fold during pregnancy, to be 100-fold greater than non-pregnant values. Exosomes present in maternal plasma may be derived from either maternal and/or fetal (placental) origin. To address this question, plasma exosomes were further characterised by the presence of a syncytiotrophoblast-specific marker (PLAP). PLAP was not detectable in the exosomal fraction from non-pregnant plasma and is consistent with previously published data [34]. Plasma exosomal PLAP displayed a similar gestational age profile to that observed for exosome number and was significantly correlated with exosomal protein and CD63 at delivery.

The syncytiotrophblast is in direct contact with maternal blood and, with a surface area of > 10 m^2^ [35], it represents a significant source of placenta vesicular membrane entering maternal blood. More that 3 g of syncytiotrophblast-derived membrane is released each day near term. The contribution of syncytiotrophblast-derived exosomes to total syncytiotrophblast membrane released, however, is not known. Similarly, no quantitative estimates of the contribution of syncytiotrophblast-derived exosomes to total maternal plasma exosomes remains to be established. The gestational variation in exosomal PLAP/CD63 data presented herein provides some estimate of the relative change in these parameters.

Exosomes mediate cell-to-cell communication and induce different effects on target cells depending on the cell origin and exosome content (*e.g*. miRNA and proteins). The function of placental-derived exosomes during normal or pathological pregnancy remains to be established. Delorme-Axfold *et al.*, [15], however, recently reported that specific miRNAs are transported in exosome released by trophoblast cells to protect the developing fetus against viral infection. In addition, we have previously reported that exosomes released from cytotrophoblast cells primary culture contain biologically active proteins [30] that can interact with the maternal endothelium and regulate their function (*e.g.* angiogenesis). Furthermore, the release of exosome from placental mesenchymal stem cells and cytotrophoblast cells is regulated by the oxygen tension [7], [30].

In this study, we used the PLAP ratio (*i.e.* immunoreactive PLAP content per exosome) as a measure of the contribution of placental exosomes to total exosomes in maternal blood. We observed that PLAP ratio was similar in both first and second trimesters but decreased dramatically in third trimester. Several factors may contribution to this decrease, including: decreased release of PLAP^+ve^ exosomes from the placenta; decreased circulating half-live of PLAP^+ve^ exosomes; increased released of PLAP^-ve^ exosomes; or a combination of these events. While it is not possible to precisely establish what causes the late gestational decreased in PLAP ratio in this study, the data afford opportunity of further hypothesis testing and, in particular, to establish the clinical utility of PLAP ratio as am indicator of placental function.

The release of placental exosomes into maternal blood may result from a change in: the *de novo* rate of secretion of exosomes; placental mass; placental perfusion; the numbers and/or surface area of syncytotrophoblasts exposed to maternal blood or a combination of these factors. In this study, the concentration of placental exosomes in maternal plasma correlated with placental mass (at delivery); and placental perfusion. The association between placental-derived exosomes and placental blood flow was assessed using Doppler velocimetry. Mean uterine pulsatility index declined gradually during pregnancy (consistent with previously published data[36]) and strongly correlated with exosomes number and PLAP concentration in maternal blood.

Exosomes isolated from peripheral plasma were biologically active, as assessed by their ability to increase endothelial cell migration *in vitro.* The bioactivity of exosomes was greatest during first trimester and gradually declined with advancing gestational age. This change in bioactivity may reflect a change in the cellular origins, and/or content of exosomes While the role of placental cell-derived exosomes in regulating maternal and/or fetal vascular responses in normal and pathological pregnancies remains to be elucidated, it is tempting to speculate that exosomes released from the syncytitotrophoblast may contribute to maternal vascular adaptation during pregnancy.

In addition to their physiological role, changes in exosome concentration and/or content may be of clinical utility in the diagnosis of placental dysfunction. Recent studies highlight the putative utility of tissue-specific nanovesicles (*e.g.* exosomes) in the diagnosis of disease onset and treatment monitoring. [37]–[42] Furthermore, the concentration of exosomal protein in plasma has been reported to increase in association with disease severity and/or progression (*e.g.* coronary heart disease), and in response to oxidative stress. [43], [44] Further studies are required to characterize the release and content of maternal peripheral plasma exosome and their diagnostic utility in pregnancies complicated by placental pathology, including pre-eclampsia, intra-uterine growth restriction and gestational diabetes.

In conclusion, during early pregnancy, the concentration of exosomes in maternal blood increase dramatically. These exosomes are biologically active and regulated endothelial cell migration. Exosomes concentration continues to increase during pregnancy, however, the contribution of placental exosomes to total plasma exosomes and exosomes bioactivity decline in late pregnancy. The data obtained in this study, for the first time, characterise longitudinal changing in maternal peripheral plasma exosomes and, as such, they provide a baseline that will facilitate comparison with complicated pregnancies and assessment of the diagnostic utility.

## Supporting Information

Figure S1 Relationship between number of exosome vesicles (NEV) and exosomal protein concentration across normal pregnancy. NEV were correlated to protein concentration for each exosome isolation Lineal correlation (-) and 95% confidence interval (—).
(TIF)
