# Supplementary figures and images for: A Gestational Profile of Placental Exosomes in Maternal Plasma and Their Effects on Endothelial Cell Migration
Source: PLoS One. 2014 Jun 6;9(6):e98667. doi: 10.1371/journal.pone.0098667 (PMC4048215; doi:10.1371/journal.pone.0098667)

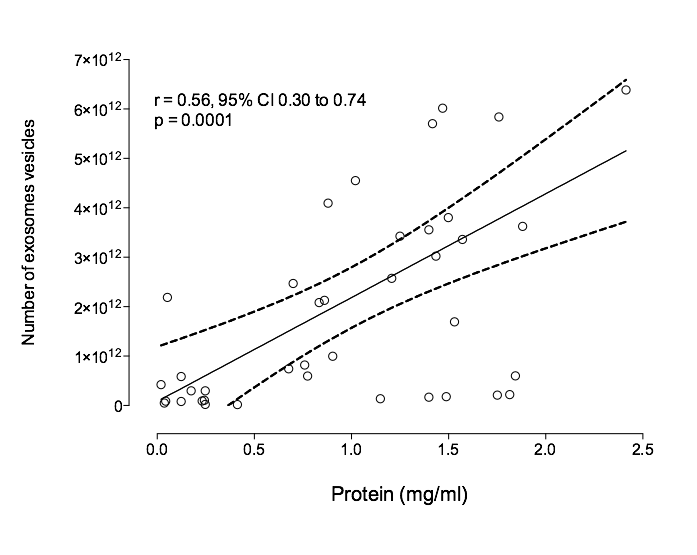

Supplement: Figure S1 — Relationship between number of exosome vesicles (NEV) and exosomal protein concentration across normal pregnancy. NEV were correlated to protein concentration for each exosome isolation Lineal correlation (-) and 95% confidence interval (—). (TIF) [file pone.0098667.s001.tif]
